# Chasing the Drop: Miami Versus Dual Criteria for Intraoperative Parathyroid Hormone Monitoring in Primary Hyperparathyroidism

**DOI:** 10.7759/cureus.96720

**Published:** 2025-11-12

**Authors:** Senthil Rajamanickam, Anurag Agarwal, Shruti Senthilkumar, Dimitrios Chatzoudis, Rachel Curran, Vignesh Balasubaramaniam, Shon Williams

**Affiliations:** 1 Otolaryngology, Head, and Neck Surgery, Betsi Cadwaladr University Health Board, Bangor, GBR; 2 General Surgery, Betsi Cadwaladr University Health Board, Bangor, GBR; 3 Otolaryngology, Betsi Cadwaladr University Health Board, Bangor, GBR; 4 Surgery, Betsi Cadwaladr University Health Board, Bangor, GBR

**Keywords:** dual criteria, miami criteria, parathyroid adenoma, parathyroidectomy, primary hyperparathyroidism

## Abstract

Aim

To compare the diagnostic performance of the Miami criterion and the Dual criterion for intraoperative parathyroid hormone (IOPTH) monitoring in patients undergoing parathyroidectomy for primary hyperparathyroidism, and to assess factors contributing to operative failure.

Methodology

A single-center retrospective observational study was conducted involving 23 consecutive patients who underwent parathyroidectomy with IOPTH monitoring between January 2023 and June 2025. The Miami criterion (≥50% parathyroid hormone (PTH) reduction from baseline at 10 minutes) and the Dual criterion (≥50% reduction with final PTH in the normal range) were applied. Operative success, defined as normocalcemia at three months, served as the gold standard. Diagnostic performance was assessed by sensitivity, specificity, positive predictive value (PPV), negative predictive value (NPV), and accuracy.

Results

At three months, 19 of 23 patients (82.6%) achieved normocalcemia. The Miami criterion demonstrated higher sensitivity (89.5%) and accuracy (78.3%) compared with the Dual criterion (78.9% and 69.6%, respectively). Both criteria showed high PPV (85.0% vs. 83.3%). Four operative failures (17.4%) were attributed to parathyroid carcinoma with metastases, non-localizing or ectopic adenomas, and vitamin D deficiency.

Conclusions

Both Miami and Dual IOPTH criteria reliably predicted postoperative normocalcemia, with the Miami criterion demonstrating higher sensitivity and accuracy. Operative failures were related to disease complexity rather than biochemical criteria. Advanced localization strategies and metabolic optimization should be integrated into perioperative planning to improve outcomes.

## Introduction

Primary hyperparathyroidism (PHPT) is the most common cause of hypercalcemia in the outpatient setting and is most frequently attributable to a solitary parathyroid adenoma, accounting for approximately 80%-85% of cases [1]. Surgical excision of the hyperfunctioning gland remains the only definitive treatment and is associated with high cure rates and durable biochemical control [2]. Traditionally, bilateral neck exploration with identification of all four parathyroid glands was considered the gold standard for parathyroidectomy, ensuring accurate diagnosis and minimizing persistent disease [3].

PHPT is one of the most common causes of hypercalcemia in the community, with its detection increasing due to widespread biochemical screening. In the United Kingdom, the reported incidence of PHPT ranges between 40 and 60 cases per 100,000 person-years, with a point prevalence approaching 0.8%, according to recent population-based data from Scotland. Earlier regional studies have documented rising incidence trends over recent decades, largely reflecting increased testing and improved diagnostic awareness [4,5]. National data from England and Wales show parathyroidectomy rates increasing from approximately 3.3 per 100,000 population in 2000 to 5.8 per 100,000 in 2010, mirroring greater recognition and surgical management of PHPT [6]. Although the true disease burden may vary across regions and study periods, these figures underscore the clinical relevance of optimizing diagnostic and intraoperative strategies for effective management of PHPT.

Over the past two decades, advances in preoperative localization and the introduction of intraoperative adjuncts have enabled a paradigm shift toward focused, minimally invasive parathyroidectomy in appropriately selected patients [7]. Among these innovations, intraoperative parathyroid hormone (IOPTH) monitoring has been particularly transformative. Given its short half-life of approximately 3 to 5 minutes, a rapid decline in parathyroid hormone (PTH) levels after excision of the hyperfunctioning gland provides real-time biochemical confirmation of cure, reducing the need for bilateral exploration and facilitating a targeted surgical approach [8-11].

The use of IOPTH monitoring has been associated with shorter operative time, lower complication rates, and improved cure rates, even in patients with multigland disease or inconclusive preoperative imaging [12]. Nevertheless, practice patterns and protocols remain heterogeneous across institutions, and outcome data from smaller centers are relatively limited [13,14]. IOPTH monitoring provides rapid biochemical confirmation of successful gland excision in PHPT. The Miami criterion, a ≥50% decline in PTH from the highest pre-excision value at 10 minutes, remains the most widely adopted protocol for predicting postoperative normocalcemia. The Dual criterion, which requires both a 50% reduction from baseline and a return of PTH to within the normal reference range, represents a more stringent approach that may improve specificity but potentially increase false-negative results [8,11]. Comparative data between these two approaches in real-world surgical practice remain limited, particularly in district general hospital settings.

In this single-center study, we evaluated the accuracy and clinical utility of IOPTH monitoring in a consecutive cohort of 23 patients undergoing surgery for PHPT. The primary objective was to assess the diagnostic performance of IOPTH monitoring in predicting surgical cure, using the Miami and Dual criteria for intra-operative decision-making, and to determine the rate of postoperative biochemical success, defined as the achievement of normocalcemia following surgery. The secondary objectives were to compare the sensitivity, specificity, positive predictive value (PPV), negative predictive value (NPV), and overall accuracy of the two criteria, and to analyze perioperative and short-term biochemical outcomes in this patient population.

## Materials and methods

Study design and patient selection

We conducted a single-center, retrospective observational study that included 23 consecutive patients who underwent parathyroidectomy for biochemically confirmed PHPT between January 2023 and June 2025. The diagnostic and management pathway followed the National Institute for Health and Care Excellence (NICE) guideline NG132 [15]. Patients were referred from primary care when presenting with persistent hypercalcemia, defined as an adjusted serum calcium level of 2.6 mmol/L or higher, in the presence of an inappropriately elevated or non-suppressed parathyroid hormone level. Secondary care evaluation included biochemical confirmation of PHPT, assessment of Vitamin D and Renal Function, exclusion of familial hypocalciuric hypercalcemia, bone mineral density measurement with dual-energy X-ray absorptiometry (DXA), and renal ultrasonography to evaluate for nephrolithiasis.

Surgical indications were based on international consensus guidelines and included symptomatic disease, albumin-adjusted calcium levels of 2.85 mmol/L or higher, evidence of end-organ involvement, or pregnancy planning [1,2,7]. All operations were performed by experienced surgeons in a District General Hospital.

Preoperative imaging and localization

A structured, stepwise approach to localization imaging was followed. High-resolution cervical ultrasonography and technetium-99m sestamibi scintigraphy were used as first-line modalities. When these studies yielded negative or discordant findings, further localization was performed with contrast-enhanced four-dimensional (4D) computed tomography (CT). In patients with recurrent hyperparathyroidism or previous failed parathyroid surgery, ¹⁸F-fluorocholine positron emission tomography (PET) was used as a second-line functional imaging technique. Focal uptake on ¹⁸F-fluorocholine PET corresponding to a discrete lesion on low-dose CT was considered indicative of a parathyroid adenoma. Several studies have demonstrated that this imaging modality has high sensitivity and specificity in reoperative and complex cases, improving surgical precision and minimizing the need for extensive exploration [16,17].

Intraoperative PTH monitoring protocol

All patients underwent IOPTH monitoring using a rapid immunoassay. Blood samples were obtained from a peripheral intravenous line at standardized intervals. Baseline samples were collected before skin incision and before excision of the gland. Post-excision PTH levels were measured at 5 and 10 minutes, with additional measurements at 15 or 20 minutes when required.

Two validated criteria were used to guide intraoperative decision-making. The Miami criterion was defined as a decrease of at least 50% in the PTH level from the highest pre-incision or pre-excision value measured 10 minutes after gland excision. The Dual criterion required a reduction of at least 50% in the PTH level from the pre-incision baseline, combined with a final PTH level within the normal reference range at 10 minutes post-excision. If the intraoperative findings and biochemical results were concordant, and either criterion was met, the procedure was concluded without bilateral neck exploration. Both criteria have been extensively validated in the literature and are associated with long-term cure rates exceeding 97% to 99% in appropriately selected patients [8,9].

Definition of operative success

The gold standard for operative success was defined as sustained normocalcemia at three months postoperatively, confirmed on at least two separate serum calcium measurements as per local protocol. Persistent disease was defined as failure to achieve normocalcemia after surgery, and recurrence was defined as hypercalcemia occurring after an initial normocalcemic interval [1,11].

Outcome measures

The primary outcome of this study was the sensitivity of the Miami and Dual intraoperative PTH criteria in predicting operative success. Sensitivity was defined as the proportion of patients who achieved normocalcemia at three months and were correctly identified intraoperatively as having achieved biochemical cure.

Secondary outcomes included specificity, PPV, NPV, and overall diagnostic accuracy of each criterion. Specificity was defined as the ability of each criterion to correctly identify patients with operative failure. PPV represented the probability that a patient identified as cured intraoperatively achieved postoperative normocalcemia, while NPV represented the probability that a patient identified as a failure intraoperatively had persistent hypercalcemia. Accuracy was defined as the proportion of correct predictions, including both true positives and true negatives, among all patients evaluated.

Statistical analysis

For each patient, intraoperative predictions based on the Miami and Dual criteria were compared with the postoperative biochemical outcome. Patients were classified as true positive, false positive, false negative, or true negative depending on the correspondence between intraoperative results and the gold standard. Sensitivity, specificity, accuracy, PPV, and NPV were calculated using standard diagnostic formulas. Categorical variables were expressed as absolute numbers and percentages. Statistical analyses were performed using SPSS version 26 (IBM Corp., Armonk, NY).

Ethics statement

The study was approved by the Institution Clinical Audit Team (Project ID: 2374). Given its retrospective design, informed consent was waived. All data were anonymized before analysis. As this was an audit of existing practice, no changes were made to clinical care pathways.

## Results

Patient characteristics

A total of 23 patients underwent parathyroidectomy with IOPTH monitoring. The mean age was 63.74 ± 12.52 years, and 15 patients (65.2%) were females. The mean three months postoperative adjusted serum calcium level was 2.57 ± 0.38 mmol/L, and the mean preoperative highest PTH level was 17.57 ± 9.61 pg/mL, and the three months postoperative highest PTH level was 7.60 ± 7.64 pg/mL.

Preoperative imaging correctly localized a lesion in 21 patients (91.3%). Ten patients underwent ultrasonography and were correctly localized in 8 patients (80%). Sestamibi scintigraphy was carried out in 23 patients and was correctly localized in 21 patients (91.3%) when compared with the histological findings. Two patients (8.7%) required ¹⁸F-fluorocholine PET/CT for localization following inconclusive conventional imaging.

Operative findings and outcomes

**Figure 1:**
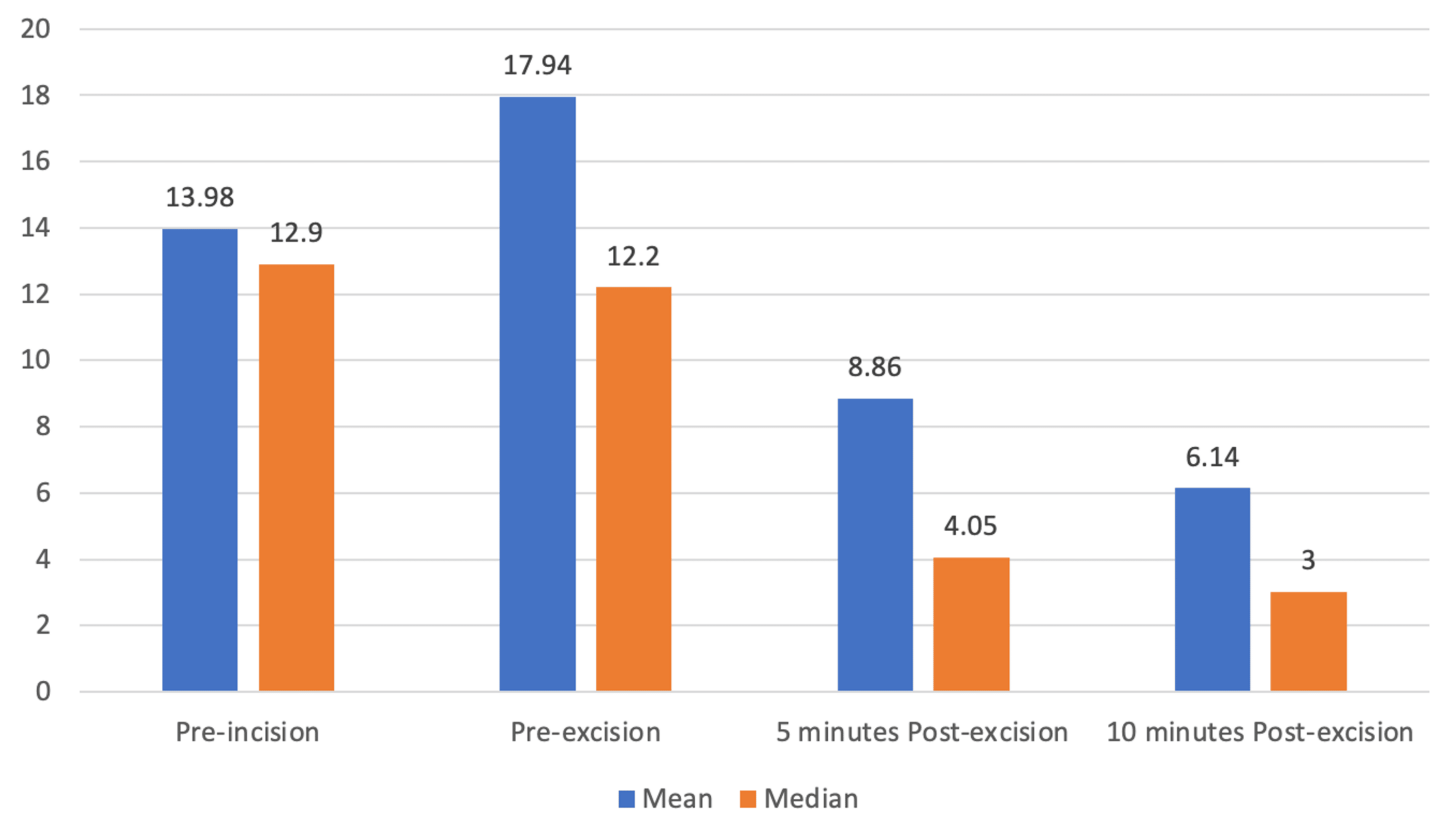
Trend in intraoperative parathyroid hormone levels. This bar chart depicts the mean and median IOPTH levels at four key time points during parathyroidectomy: pre-incision, pre-excision, 5 minutes post-excision, and 10 minutes post-excision. A progressive decline in IOPTH levels is observed following excision of the hyperfunctioning gland. The corresponding median values show a similar downward trend. This pattern reflects the expected rapid drop in circulating PTH following successful removal of the hypersecreting gland, supporting its use as a real-time intraoperative marker of biochemical cure. Normal range of PTH: 1.6-6.9 pmol/L. PTH, parathyroid hormone; IOPTH, intraoperative parathyroid hormone

A single parathyroid adenoma was identified in 19 patients (82.6%), parathyroid hyperplasia in 3 patients (13%), and parathyroid carcinoma in 1 patient (4.4%). All procedures were completed using a focused or minimally invasive approach. There were no intraoperative complications. The IOPTH serum trend is depicted in Figure 1.

At three months postoperatively, 19 of 23 patients (82.6%) achieved sustained normocalcemia, meeting the predefined gold standard for operative success. Four patients (17.4%) had persistent hypercalcemia, one of whom was correctly identified intraoperatively as a failure.

Diagnostic performance of the IOPTH criteria

**Figure 2:**
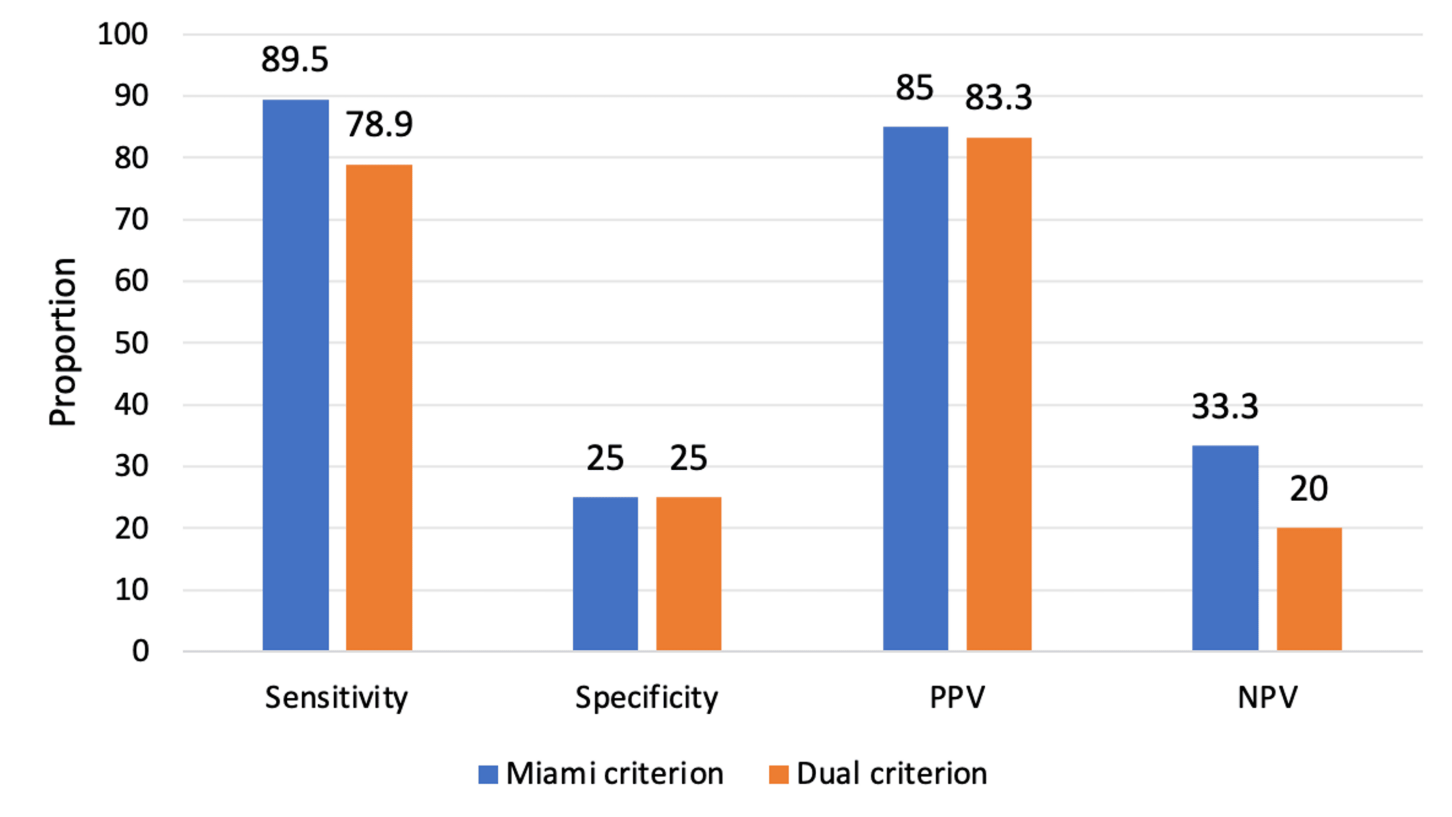
Comparison of diagnostic performance between Miami and Dual IOPTH criteria. This bar chart illustrates the diagnostic performance of the Miami and Dual IOPTH monitoring criteria in predicting postoperative biochemical cure in patients undergoing surgery for primary hyperparathyroidism. The Miami criterion demonstrated higher sensitivity (89.5%) and accuracy (78.3%) compared with the Dual criterion (78.9% and 69.6%, respectively). Both criteria showed comparable positive predictive values (85.0% for Miami vs. 83.3% for Dual). PPV, positive predictive value; NPV, negative predictive value; IOPTH, intraoperative parathyroid hormone

When applying the Miami criterion, 17 patients were true positives, 3 were false positives, 2 were false negatives, and 1 was a true negative. This corresponded to a sensitivity of 89.5%, specificity of 25%, PPV of 85%, NPV of 33.3%, and overall accuracy of 78.3%, as depicted in Figure 2.

When applying the Dual criterion, 15 patients were true positives, 3 were false positives, 4 were false negatives, and 1 was a true negative. This yielded a sensitivity of 78.9%, specificity of 25%, PPV of 83.3%, NPV of 20%, and overall accuracy of 69.6%, as depicted in Figure 2. Although sensitivity and accuracy were numerically higher with the Miami criterion than with the Dual criterion, specificity was low for both criteria, reflecting the small number of operative failures.

Postoperative course and complications

The mean serum calcium level at three months was 2.57 ± 0.38 mmol/L in patients with successful surgery. No patients developed permanent hypocalcemia or recurrent laryngeal nerve injury. One patient underwent successful re-exploration for persistent disease.

At three months postoperatively, 19 of 23 patients (82.6%) achieved sustained normocalcemia, meeting the predefined gold standard for operative success. Four patients (17.4%) experienced persistent hypercalcemia, representing operative failure. The contributing factors in these cases were clinically significant.

One patient was subsequently diagnosed with parathyroid carcinoma with chest metastases on follow-up imaging. This patient had undergone one previous parathyroidectomy. A second patient had a non-localizing postoperative Single-Photon Emission Computed Tomography (SPECT) sestamibi scan. A third patient had a non-localizing postoperative SPECT, but ¹⁸F-fluorocholine PET/CT later identified a contralateral adenoma, possibly located in a paraesophageal position. The fourth failure was attributed to dual pathology with vitamin D deficiency, likely contributing to persistent biochemical abnormalities despite resection. These cases highlight the heterogeneity of operative failure etiologies, including malignant disease, reoperative necks, non-localizing or challenging adenomas, and coexisting metabolic pathology.

## Discussion

In this single-center study of 23 patients undergoing parathyroidectomy for PHPT, we compared the diagnostic performance of two well-validated IOPTH monitoring protocols: the Miami criterion and the Dual criterion. The Miami criterion demonstrated a higher sensitivity (89.5%) and overall accuracy (78.3%) than the Dual criterion (sensitivity 78.9%, accuracy 69.6%). Both criteria exhibited high PPVs (85.0% and 83.3%, respectively), supporting their role as reliable intraoperative adjuncts in predicting postoperative normocalcemia. These findings are consistent with previous large series demonstrating sensitivities of 85%-95% and accuracies approaching 90% for the Miami criterion, and slightly lower performance for the Dual criterion [9,10,13]. The low specificity observed for both protocols likely reflects the small number of operative failures in this cohort rather than limitations of the criteria themselves.

The Miami criterion offers several practical advantages. It is based on a single time point at 10 minutes following gland excision, enabling efficient intraoperative decision-making and reducing operative time. This is particularly advantageous in centers performing focused or minimally invasive procedures, where rapid confirmation of biochemical cure can obviate the need for bilateral exploration. In contrast, the Dual criterion applies a more stringent threshold, which may increase the likelihood of false-negative results in patients with slower PTH decay kinetics. This phenomenon has been described in patients with large adenomas or markedly elevated baseline PTH levels, in whom physiological decline may lag behind the 10-minute sampling window [9,18]. In our study, two patients were misclassified by the Dual criterion but ultimately achieved postoperative normocalcemia, illustrating this limitation.

Both protocols demonstrated high PPVs, reinforcing their role in modern parathyroid surgery. Numerous studies have confirmed that intraoperative PTH monitoring improves operative precision, facilitates minimally invasive approaches, and allows targeted excision without compromising long-term cure rates [9,10,12]. Our findings align with this evidence and support the continued use of intraoperative monitoring as a standard adjunct in focused parathyroidectomy.

Importantly, persistent hypercalcemia occurred in four patients (17.4%), reflecting the complexity of certain disease scenarios rather than failure of intraoperative biochemical monitoring itself. One patient had parathyroid carcinoma with subsequent metastatic disease, an uncommon but recognized cause of persistent or recurrent hypercalcemia that often requires multimodal management and carries a less favorable prognosis. Another patient had undergone one previous parathyroidectomy. Reoperative parathyroid surgery is well known to have lower cure rates and increased difficulty, owing to distorted anatomy, scar tissue, and higher rates of multigland disease.

Two failures were directly related to localization challenges. In one patient, neither postoperative SPECT sestamibi nor ¹⁸F-fluorocholine PET/CT identified the residual disease. In another, a contralateral paraesophageal adenoma was detected only on postoperative ¹⁸F-fluorocholine PET/CT. These findings underscore the critical role of advanced functional imaging in detecting elusive or ectopic adenomas, particularly in reoperative or anatomically complex cases. Several studies have demonstrated the superior sensitivity of ¹⁸F-fluorocholine PET/CT compared with conventional imaging in these settings [16,17,19].

The fourth failure was associated with dual pathology and vitamin D deficiency. Vitamin D deficiency is a well-recognized confounder in the management of PHPT and can contribute to persistently elevated PTH levels even after resection of a hyperfunctioning gland. This can complicate intraoperative interpretation and postoperative biochemical follow-up, highlighting the importance of preoperative optimization of metabolic parameters to minimize diagnostic uncertainty and improve outcomes [20,21].

Taken together, these four cases emphasize that while IOPTH monitoring is highly predictive of cure in straightforward cases, persistent disease is more likely in patients with parathyroid carcinoma, prior neck surgery, challenging adenoma localization, or coexisting metabolic abnormalities. These factors, rather than intraoperative criteria alone, often determine the ultimate success of surgery. This aligns with prior reports indicating that reoperative cases and malignant or multi-gland disease remain the primary drivers of persistent hyperparathyroidism despite modern surgical and biochemical advances [9,10].

From a management perspective, persistent hyperparathyroidism requires an individualized and multidisciplinary approach. For patients with localization challenges, repeat imaging with ¹⁸F-fluorocholine PET/CT or alternative functional modalities may facilitate targeted reoperation. Reoperative surgery should ideally be performed in experienced hands, given the increased technical difficulty and risk of complications. In patients with parathyroid carcinoma, long-term surveillance and multidisciplinary input are essential, with consideration of resection of metastatic foci or systemic therapy when appropriate. In cases of dual pathology, including vitamin D deficiency, correction of metabolic abnormalities before and after surgery may improve outcomes and reduce the risk of biochemical misinterpretation.

Despite these complexities, the overall high PPVs observed for both criteria confirm their value in guiding intraoperative decision-making. The Miami criterion, in particular, remains a practical and reliable tool for achieving biochemical cure in the majority of patients.

Strengths and limitations

Our study has several strengths, including the use of standardized IOPTH protocols, uniform follow-up at three months, and systematic application of multimodal imaging. However, its limitations include the modest sample size, which limits statistical power, and the low number of operative failures, which affected specificity and NPV calculations. The single-center design may also limit generalizability to lower-volume institutions or those with differing perioperative protocols. Given the limited number of eligible patients, this study is presented as an initial single-center experience, intended to contribute to future multicenter research.

## Conclusions

In this single-center cohort of 23 patients, both the Miami criterion and the Dual criterion for IOPTH monitoring demonstrated high PPVs in predicting postoperative biochemical cure following parathyroidectomy for PHPT. The Miami criterion showed higher sensitivity and overall diagnostic accuracy, supporting its use as a practical and reliable intraoperative tool for guiding focused parathyroid surgery. Four cases of operative failure were observed, all attributable to factors beyond intraoperative PTH criteria, including parathyroid carcinoma with metastatic disease, previous extensive neck surgery, non-localizing or anatomically challenging adenomas, and vitamin D deficiency. These findings highlight that the effectiveness of intraoperative biochemical monitoring depends not only on the criterion applied but also on accurate preoperative localization, optimization of metabolic parameters, and recognition of complex or atypical disease patterns. Surgeons should incorporate advanced functional imaging and individualized perioperative planning to maximize cure rates. Larger multicenter studies are warranted to refine the optimal application of intraoperative PTH criteria in complex and reoperative settings.
